# The influence of self-esteem and self-efficacy on doping susceptibility and intention in amateur athletes and exercisers

**DOI:** 10.3389/fspor.2026.1783309

**Published:** 2026-04-22

**Authors:** Diana Korinna Zazueta-Beltrán, Roxana Abril Morales-Beltrán, Karla Noelia Cruz-Morales, Hussein Muñoz-Helú, Javier Arturo Ríos-Mena, Luis Felipe Reynoso-Sánchez

**Affiliations:** 1 Department of Health Sciences, Autonomous University of Occident, Los Mochis, Mexico; 2 Department of Social Sciences and Humanities, Autonomous University of Occident, Los Mochis, Mexico; 3 Department of Economic-Administrative Sciences, Autonomous University of Occident, Los Mochis, Mexico; 4 Research Centre for Physical Culture Sciences and Health, Autonomous University of Occident, Culiacán, Mexico; 5 Faculty of Psychology, Autonomous University of Nuevo Leon, Monterrey, Mexico

**Keywords:** body image, competence-related self-belief, doping behavior, performance enhancement substances, protective psychological factors, self-concept

## Abstract

**Background:**

Doping is a practice that poses a risk to the physical and mental health of those who exercise. Psychological variables such as self-esteem and self-efficacy have been shown to influence the intention to dope. The objective was to analyze the mediating effect of self-esteem and self-efficacy on susceptibility to doping in amateur athletes and exercisers.

**Method:**

This study surveyed 392 participants who exercised regularly. It was a cross-sectional study with a descriptive correlational scope, and sequential mediation analysis. The assessment was based on the World Anti-Doping Agency (WADA) research package scales of self-esteem, self-efficacy, intention, and susceptibility to doping and was supported by the Drug Control Model in Sport.

**Results:**

The results indicate that the mediation model was approved, based on the inference that susceptibility to doping is positive associated with the intention to dope and, in this case, this susceptibility is mediated by self-esteem and self-efficacy. The direct effect (0.447) is slightly greater than the indirect effect (0.346), both of which are significant. These values indicate partial mediation: that is, susceptibility to doping is related to higher intention to dope, but when it passes through self-esteem and self-efficacy, its relationship is reduced.

**Conclusion:**

These findings support the hypothesis that psychological variables can act as intermediate mechanisms in the relationship between susceptibility to doping and intention to dope. This suggests the need to increase preventive strategies that promote psychoeducation of psychological variables such as self-esteem and self-efficacy, which reinforce the protective factor against pro-doping behavior in athletes.

## 1 Introduction

Sport, as a universal cultural phenomenon, aims to foster human development by focusing on strengthening the biopsychosocial capacities of participants (1).This aligns with the idea that sporting environments support physical and mental health, as well as the growth of educational settings governed by systems that promote ethical principles and sportsmanship (2).

However, modern sports history has revealed incidents involving athletes whose behavior violates sporting ethics and self-care, showing morally and regulatory inappropriate actions aimed at gaining advantages to achieve outcomes that fulfill their personal interests, social recognition, and sporting prestige (3–5).These actions are closely connected to pro-doping tendencies within sports contexts (6, 7).

According to the World Anti-Doping Agency (8), doping involves using prohibited substances and/or methods to improve physical performance in ways that break sport rules. Such actions not only hurt the athlete's public image but also harm their overall health (9), moving this issue beyond just performance and sports ethics, and into athlete health self-care.

Although historically the use of substances or methods banned by WADA has been viewed as an issue only among elite athletes, institutions such as the European Commission (10) have observed that doping is not restricted to high-performance sports. It also happens in amateur and recreational sports (11), and among individuals who exercise for health or appearance (an increasingly concerning trend) (12).

The prevalence of prohibited substances or methods used among recreational athletes varies widely in literature, making it difficult to draw definitive conclusions. For example (13), reported a 18.3% prevalence of controlled substance use for performance and appearance enhancement in European populations. Meanwhile (14), found a 1.6% prevalence of doping-related substance use and a 10.6% use of over-the-counter performance enhancers that may contain doping agents. Although these figures seem low, specific substances such as anabolic-androgenic steroids show much higher rates, exceeding 41.67% (15) and 47.45% (16). In Latin American populations, evidence remains limited, with reports indicating a 3.4% prevalence of current doping substance use and 4.3% intention to use them in the future (17). However, it is reasonable to assume that countries with fewer technical, infrastructural, or educational resources may face greater challenges in identifying consumption in recreational settings and detecting doping cases in sport (18).

Ultimately, doping in amateur athletes or exercisers is a concerning issue. In competitive sports, detection methods and protocols pose challenges for effective enforcement (19), while in amateur sports, the widespread use of substances and nutritional supplements can be harmful due to poor regulation and insufficient guidance (11, 20). This situation is further worsened by a commercial market with confusing marketing and lacking regulatory oversight, allowing purchases without expert advice.

### 1.1 Psychosocial risks and repercussions associated with doping

The importance of this issue among amateur athletes and exercisers lies in the physical, psychological, social, and contextual risks it involves (4). Physically, using ergogenic substances can lead to dependence, addiction, and cardiovascular or endocrine changes (11). Similarly, taking supplements without professional guidance may add extra health risks (19, 21).

Psychologically, several studies have found links between doping substance use and mood-related issues, such as depressive symptoms (22), as well as psychopathic traits, irritability, and impulsivity (23). Socially, doping may cause conflicts, stigmatization, and loss of recognition within the sporting community (9).

Additionally, factors such as social pressure, competitive demands, and ambiguous norms can promote the adoption of these practices (24, 25). Some athletes may even rationalize unethical behaviors (such as deception or cheating) (26) or justify doping as a shortcut to improve performance or physical appearance (27). These processes are often reinforced by mechanisms such as the false social consensus supporting doping (5) and moral disengagement, which allows individuals to justify ethically wrong actions (28).

### 1.2 Self-esteem and self-efficacy in doping susceptibility and intention

Doping susceptibility in elite athletes has been linked to the desire for social recognition that is common in ego-oriented motivational climates, as well as unhealthy levels of self-esteem and self-efficacy (28, 29). Among recreational athletes, motivations more often focus on performance improvement, health, or physical appearance (30), although the underlying psychological mechanisms are similar. In this group, the pressure to improve results may increase the likelihood of using unsupervised supplements (31), or even doping substances (15–17).

The literature agrees that doping intention and susceptibility are complex phenomena in which self-esteem and self-efficacy serve as important predictors (29, 32, 33). From Branden's (34) perspective, self-esteem is a state of personal satisfaction resulting from self-actualizing experiences, and its presence is linked to better sports outcomes, increased well-being, and protective attitudes against forbidden substance use (34, 35).

Strong self-esteem boosts traits like self-confidence, self-regulation, and responsible decision-making (36, 37), helping build emotional resilience when facing defeats, setbacks, or frustration. Conversely, low self-esteem can make individuals more vulnerable to justifying pro-doping behaviors, especially in high-pressure situations (38, 39).

Self-efficacy, meanwhile, has become a key factor in physical and sports behavior (40–42). According to Bandura (43), it involves beliefs about one's ability to organize and carry out actions needed to reach goals. Reduced self-efficacy may lead to negative expectations and unhelpful decisions, while higher levels encourage ethical choices, self-control, and resistance to situational pressures (43–45). Strengthened self-efficacy is also linked to greater moral strength and less acceptance of pro-doping messages, decreasing moral justification and cognitive strategies that legitimize such behaviors (29, 46).

### 1.3 Explanatory frameworks on how psychological factors influence doping susceptibility and intention

A theoretical approach that helps illustrate how psychological factors relate to doping is Bandura's (43) Social Cognitive Theory, which suggests that perceived self-efficacy dynamics are shaped by: 1) The personal component, referring to belief systems and self-control strategies that enable resisting temptation to use enhancement substances and overcoming risks. Conversely, when perceived incapacity dominates, doping intention increases. 2) The environmental component, which includes social pressures, norms, and the influence of models within sport culture. When environmental demands coincide with unclear norms and permissive models, susceptibility to doping rises. 3) The behavioral component, related to decision-making. Individuals with high self-esteem and self-efficacy are less afraid of what must be done to reach goals, while those with low levels see tasks as more complicated, hindering effort and achievement, making “easy ways” or “helps” such enhancing substances more attractive to use.

In the context of Social Cognitive Theory, doping is intentional, self-regulated behavior influenced by beliefs, expectations, and self-assessment processes (47). Although efforts have been made to study psychological variables such as self-esteem and self-efficacy in relation to doping susceptibility and intention, the field remains underexplored (32), especially among amateur or recreational athletes. In this context, it is scientifically and socially important to examine the role of self-efficacy (46) and self-esteem (24), as low levels of these variables are associated with greater doping intent and susceptibility. Self-efficacy has been shown to be a protective factor, since when athletes have a strong self-confidence system (personal component), they must train effectively, improve their performance naturally, and are able to resist social pressure, which reduces the perception of the need to use substances to improve performance and the intention to dope (29, 48). On the other hand, direct measures of global self-esteem in relation to doping intention are scarce, but, some studies based on social cognitive ideas have been observed (49, 50), whose objective was to improve self-esteem and self-management as protective resources, but the primary results refer directly to self-efficacy and the regulation of emotions associated with doping prevention.

**Figure 1:**
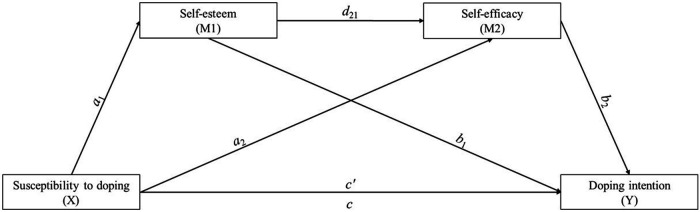
Sequential mediation model diagram *X* → *M*1 → *M*2 → *Y*.

In the proposed theoretical model (Figure 1), self-esteem is positioned as a predictor of self-efficacy because it represents a global self-evaluation that functions as an affective-cognitive framework through which personal abilities are interpreted. According to Bandura (43) and hierarchical models of self-concept, efficacy beliefs are built on broader self-evaluative processes. Therefore, higher self-esteem supports competence-related interpretations, boosting self-efficacy, while low self-esteem tends to diminish it.

### 1.4 Study purpose

To contribute to expanding knowledge about a growing phenomenon and, subsequently, to developing intervention strategies that help reduce the incidence of such behaviors among exercisers, this study aimed to analyze the mediating effects of self-esteem and self-efficacy on doping susceptibility and doping intention in amateur athletes and exercisers.

Susceptibility to doping in amateur athletes and exercisers increases the risk of consuming prohibited substances to enhance their performance (*X*), preceding the intention to dope (*Y*). Therefore, it will be verified whether psychological variables such as self-esteem (*M*_1_) and self-efficacy (*M*_2_) can mediate the reduction of this risk in amateur athletes and exercisers, as these variables strengthen the ability to decide not to use prohibited substances to gain a competitive advantage or improve physical appearance.

In this proposed model, we examine how the prediction of the independent variable susceptibility to doping (*X*) relates to the dependent variable intention to dope (*Y*), with self-esteem (*M*_1_) and self-efficacy (*M*_2_) acting as mediating variables that facilitate the connection between the stimulus and the response of the same (51). These mediating variables are essential in defining the relationship between the predictor variable (*X*) and the criterion variable (*Y*). The potential causal relationship between *X* and *Y* is described by this scheme: *X* → *M*_1_ → *M*_2_ → *Y*.

In order to verify whether other demographic characteristics such as gender, age, years of experience, level of practice, and academic level influence the intention to dope, mediated by self-esteem and self-confidence, the study set specific objectives to describe the association of each of these variables as an independent variable (*X*) on the intention to dope (*Y*), through the sequential mediation mechanism of self-esteem (*M*1) and self-efficacy (*M*2).

Based on empirical evidence regarding the relationship between self-esteem and self-concept on doping intention, as well as support from Bandura's theory (43), the following study hypotheses are proposed: 1) Self-esteem and self-efficacy will jointly mediate the relationship between doping susceptibility and doping intention among amateur athletes and exercisers. 2) Self-esteem will mediate the relationship between doping susceptibility and doping intention. 3) Self-efficacy will mediate the relationship between doping susceptibility and doping intention.

## 2 Materials and methods

### 2.1 Study design

This research used a quantitative approach with a cross-sectional, correlational design that included mediation analyses. The study is part of a larger project exploring attitudes toward nutritional supplements, performance-enhancing substances, and their connection with psychological variables among individuals who participate in physical exercise. The project received approval from the Research Ethics Committee of the Universidad Autonoma de Occidente, Mexico (CM-UAdeO 26.01/2024).

### 2.2 Sampling

Two non-probabilistic sampling strategies were employed. First, snowball sampling was utilized as an initial approach (52). A list of coaches from various locations and sporting contexts was compiled, and they were asked to assist in recruiting participants who met the inclusion criteria. This initial group referred to additional potential participants, allowing the sample to expand gradually.

Because the target sample size was not reached through this method alone, convenience sampling was used afterward as a supplementary strategy (53). These efforts included sharing the study through digital channels like social media, posting printed posters in gyms, fitness centers, and making in-person visits to clubs, sports complexes, universities, and recreational areas where athletes and exercisers were present.

#### 2.2.1 Participants

The population included amateur athletes and exercisers who met the following criteria: (a) being at least 18 years old, (b) participating in structured physical activity at least three times a week, and (c) having at least six months of experience. A total of 415 individuals completed the battery of tests. Because adherence to the inclusion criteria was crucial, data was carefully screened, resulting in a final sample of 392 participants.

Participants were aged between 18 and 79 years (*M* = 26.12, SD = 9.88), and the sample included men (*n* = 228) and women (*n* = 164). Table 1 presents the sociodemographic characteristics and the types of sports or exercise modalities practiced. The diversity of sport modalities aligns with previous quantitative studies on doping behaviors (24, 54).

**Table 1:** Demographic information of the participants.

Demographics	*f*	%
Gender		
Female	164	41.84
Male	228	58.16
Level of practice		
Amateur	228	58.16
Recreative	164	41.84
Exercise type		
Soccer	68	17.35
Track and field	25	6.38
Volleyball	28	7.14
Beisball/Softball	30	7.65
Running	20	5.10
Basketball	14	3.57
Handball	9	2.30
Cycling	9	2.30
Combat sports	14	3.57
Gym/Fitness	124	31.63
Crossfit	6	1.53
Functional trainig	10	2.55
Bodybuilding	3	0.77
Swimming	4	1.02
Padel	2	0.51
Dance	4	1.02
Hike	7	1.79
Calisthenics	4	1.02
Spinning	3	0.77
Pilates	3	0.77
Yoga	1	0.26
Years of experience		
<1 year	51	13.01
1–2 years	62	15.82
3–5 years	82	20.92
6–10 years	75	19.13
>10 years	122	31.12
Academic degree		
Secondary school	5	1.28
High school	53	13.52
Technical career	23	5.87
Bachelor’s degree	255	65.05
Post-graduate degree	56	14.29

### 2.3 Procedure

This study was conducted from September 2024 to October 2025. The stages were as follows: obtaining approval from the bioethics committee of the Universidad Autonoma de Occidente, securing funding through the Institutional Program for the Promotion of Postgraduate Research (PIFIP 2024) at that institution, collecting data using various instruments (September 2024 to January 2025), analyzing data, interpreting results, and writing the article.

The initial step involved selecting and organizing the questionnaire with the help of a group of sports psychology experts. Next, scales measuring self-esteem, self-efficacy, doping intention, and susceptibility to doping were chosen from the anti-doping research package developed by the World Anti-Doping Agency (WADA), based on the Sport Drug Control Model proposed by Donovan et al. (55).

The finalized questionnaire was digitized using the SurveyMonkey online platform. A pilot test was conducted with a group of athletes (*n* = 20) to ensure understanding of the items. During the data collection phase, each participant accessing the anonymous survey was first presented with information about the study's purpose and procedures. To proceed, individuals were required to check a verification box confirming their informed consent. These ethical considerations complied with the *Reglamento de la Ley General de Salud en Materia de Investigación para la Salud in Mexico,* which classifies the study as “research without risk,” Article 17, Section I (56). Additionally, the study was conducted in accordance with the Declaration of Helsinki (57) and followed current recommendations for research in sport and exercise sciences (58).

### 2.4 Instruments

#### 2.4.1 Doping susceptibility

Susceptibility to doping is a key focus for anti-doping agencies, as it involves estimating how likely an individual is to engage in using prohibited substances (59). In this study, susceptibility was measured using an indirect question based on a hypothetical scenario: “If you were offered a prohibited substance to improve your performance or physical appearance, under medical supervision, at low or no cost, that was currently undetectable and could make a significant difference in your performance or appearance, how seriously would you consider this offer?” This question format has proven to be more effective because it is often seen as less invasive, encouraging more honest responses from participant (55). The item is answered on a four-point Likert scale, ranging from 1 = “not at all” to 4 = “a lot.”

#### 2.4.2 Doping intention

Intention to dope was evaluated using the questionnaire adapted by (60), which includes four items presenting hypothetical scenarios where participants indicate their level of agreement on the use or consumption of doping substances. Responses are recorded on a five-point Likert scale (1 = “strongly disagree” to 5 = “strongly agree”). In this study, the scale demonstrated adequate reliability, with a Cronbach's alpha coefficient of *α* = .70.

#### 2.4.3 Self-esteem

Self-esteem was measured using the (61), validated in Mexico for young adults (62). This tool assesses perceptions of personal worth and self-esteem levels. It includes 10 items with a four-point response scale (1 = “strongly disagree” to 4 = “strongly agree”); higher scores indicate higher self-esteem. The scale has good reliability, with an internal consistency of *α* = .77.

#### 2.4.4 Self-Efficacy against doping

Self-efficacy in avoiding doping substances was evaluated using the Self-Efficacy Scale for Regulating Doping Behaviors, developed by Lucidi et al. (47). This tool assesses how much athletes believe they can avoid using forbidden substances in different risky situations. Responses are given on a seven-point Likert scale (1 = “not at all capable” to 7 = “completely capable”). The scale has shown excellent psychometric properties, with high reliability and adequate validity (29). In this study, the Cronbach *α* was 0.97.

### 2.5 Data analysis

Data processing and analysis were conducted using IBM SPSS v.27 and JASP v.0.19.3 software. First, the internal consistency of the scales was evaluated with Cronbach's alpha coefficient. Next, the distribution of the variables was checked using the Shapiro–Wilk test, with skewness and kurtosis confirming the lack of normality.

Descriptive statistics (frequencies, means, and standard deviations) were then calculated, and inferential analyses were conducted using Spearman's Rho correlations. To test the hypotheses, a sequential multiple mediation model was applied with the PROCESS v.5.0 macro (Model 6), utilizing a bootstrapping procedure with 5,000 resamples and 95% confidence interval estimates. An effect was deemed significant when the confidence interval between the lower limit (LL) and upper limit (UL) did not include zero.

## 3 Results

The results in Table 2 show that 64.0% of the participants would not be open to using a prohibited substance, even if it posed no health risks, was low cost, and was undetectable. This may mean that decisions can be influenced by psychological factors and a lack of perceived need to use substances for performance enhancement. However, 35.9% of the athletes (combining 21.4% who considered it a little, 9.7% who considered it somewhat, and 4.8% who considered it a lot) demonstrate some level of susceptibility, suggesting a willingness to consider using a substance to improve athletic performance. These results highlight a notable risk of doping susceptibility.

**Table 2:** Results of frequency levels and percentage of self-esteem, self-efficacy, and doping intention in amateur athletes and exercisers.

Variable	Level	Frequency	Percentage
Doping susceptibility	I would not consider it at all	251	64.0%
	A little consideration	84	21.4%
	Some consideration	38	9.7%
	A lot of consideration	19	4.9%
	Total	392	100%
Self-esteem	Low	43	11.0%
	Middle	58	14.8%
	High	291	74.2%
	Total	392	100%
Self-efficacy	Low	51	13.0%
	Middle	28	7.2%
	High	313	79.8%
	Total	392	100%
Doping intention	Low	216	55.1%
	Middle	136	34.7%
	High	40	10.2%
	Total	392	100%

The percentage results show that the exercisers have high levels of self-esteem and self-efficacy (74.2% and 79.8%, respectively). However, the occurrence of doping intentions in the medium and high categories (34.7% and 10.2%), totaling 44.9%, needs careful attention, as it nears 50%. This figure may indicate a tendency to engage in doping behaviors, which could harm both the athletes' health and their sports performance.

Table 3 displays descriptive results, reliability indices, and correlations related to the evaluated variables. Cronbach's alpha coefficients indicate internal consistency levels ranging from acceptable to excellent, supporting the psychometric validity of the instruments for further analysis. Spearman's Rho results show that self-esteem is negatively and significantly correlated with susceptibility to doping (*ρ* = −.203, *p* < .01) and with the intention to dope (*ρ* = −.253, *p* < .01), suggesting that higher self-esteem is linked to lower vulnerability and a lesser likelihood of intending to use prohibited substances. Conversely, self-esteem has a positive and significant correlation with self-efficacy (*ρ* = .206, *p* < .01), reflecting an expected relationship between these psychological variables.

**Table 3:** Descriptive statistics, Spearman’s rho and Cronbach’s alpha coefficients*.*

	*M*	*SD*	*α*	Skewness	Kurtosis	1	2	3
Doping susceptibility	1.33	1.04	.71	1.47	1.19	—		
Self-esteem	32.54	5.41	.71	−0.87	0.93	−.20	—	
Self-efficacy against doping	59.17	19.84	.99	−0.17	1.40	−.23**	.21**	—
Doping intention	6.49	3.29	.85	1.49	0.18	.53**	−.25**	−.34**

*M* = Mean; *SD* = Standard deviation; *α* = Cronbach Alpha; 1 = Doping susceptibility; 2 = Self-esteem; 3 = Self-efficacy against doping.

** *p* < .01 (bilateral).

Self-efficacy also demonstrated a negative and significant correlation with doping susceptibility (*ρ* = −.225, *p* < .01) and with the intention to dope (*ρ* = −.339, *p* < .01), indicating that a higher perception of personal ability is associated with a lower risk of doping. Additionally, as expected, susceptibility to doping showed the strongest positive correlation with doping intention (*ρ* = .530, *p* < .01), which allows us to observe that greater predisposition is closely linked to a higher likelihood of intending to dope.

### 3.1 Results of the multiple sequential mediation analysis

**Figure 2:**
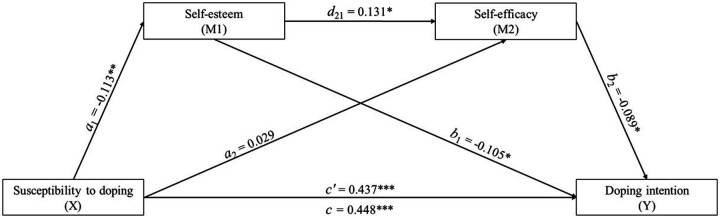
Multiple sequential mediation model illustrating the indirect effects of susceptibility to doping on doping intention through self-esteem and, subsequently, self-efficacy. Direct effect: *β* = 0.437 (LL = 0.373, UL = 0.501); Indirect effect 1: (*X* → *M*_1_ → *Y*): *a*_1_*b*_1_ = 0.011 (LL = 0.001, UL = 0.029); Indirect effect 2: (*X* → *M*_2_ → *Y*): *a*_2_*b*_2_ = −0.003 (LL = −0.012, UL = 0.005); Indirect effect 3: (*X* → *M*_1_ → *M*_2_ → *Y*): *a*_1_*d*_21_*b*_1_ = 0.001 (LL = 0.000, UL = 0.003); Total effect: *β* = 0.447 (LL = 0.383, UL = 0.511). *p < .05; **p < .01; ***p < .001.

The multiple sequential mediation model examined whether self-esteem (*M*_1_) and self-efficacy (*M*_2_) mediated the association between susceptibility to doping (*X*) and doping intention (*Y*) (Figure 2 and Table 4). The direct effect of susceptibility on doping intention was positive and statistically significant [*β* = .4477, *t* = 13.69, *p* < .001, 95% CI (0.3834, 0.5119)], explaining 32.49% of the variance in intention (*R*^2^ = .3249, *F* = 187.66, *p* < .001), indicating that athletes with higher susceptibility also exhibited stronger intentions to engage in doping.

**Table 4:** Regression coefficients and mediation summary for the sequential effects of self-esteem and self-efficacy in the relationship between doping susceptibility and doping intention.

Antecedent	*M*_1_ (Self-esteem)			*M*_2_ (self-efficacy)			*Y* (Doping intention)		
	Coef.	SE	CI 95% (*p*-value)	Coef.	SE	CI 95% (*p*-value)	Coef.	SE	CI 95% (*p*-value)
*X* (Doping susceptibility)	−0.113	0.039	−0.190, −0.035 (.004)	0.029	0.041	−0.053, 0.110 (.489)	0.437	0.033	0.373, 0.501 (<.01)
*M*_1_ (Self-esteem)				0.131	0.053	0.028, 0.234 (.013)	−0.105	0.042	−0.187, −0.023 (.012)
*M*_2_ (self-efficacy)							−0.089	0.040	−0.168, −0.010 (.027)
Constant	2.808	0.070	2.670, 2.945 (<.01)	2.280	0.164	1.957, 2.602 (<.01)	1.385	0.159	1.073, 1.697 (<.01)
	*R*^2^ = 0.021			*R*^2^ = 0.016			*R*^2^ = 0.346		
	*F* (1, 390) = 8.169, =0.004			*F* (2, 389) = 3.163, =.043			*F* (3, 388) = 68.556, <0.001		

Coef., unstandardized coefficient; SE, standard error; CI 95%, confidence intervals at 95%; Bootstrap confidence interval based on 5,000 samples.

The path from susceptibility to self-esteem (*X* → *M*_1_) was significant and negative [*β* = –.113, *t* = –2.86, *p* = .004, 95% CI (–0.190, –0.035)], indicating that higher susceptibility was linked to lower self-esteem. In contrast, doping susceptibility did not significantly predict self-efficacy (*X* → *M*_2_) *β* = .029, *t* = .69, *p* = .48, 95% CI [–0.053, 0.110]). The relationship between the mediators was significant: self-esteem positively predicted self-efficacy (*M*_1_ → *M*_2_; *β* = .130, *p* = .013), suggesting that athletes with higher self-esteem also reported greater self-efficacy.

Both mediators were significant negative predictors of doping intention. Higher self-esteem was linked to a lower intention to dope [*β* = –.105, *t* = –2.51, 95% CI (–0.187, –0.023)], as was greater self-efficacy [*β* = –.089, *t* = –2.22, 95% CI (–0.168, –0.010)]. This suggests that these variables could be associated with a greater capacity to respond to the intention to dope.

Concerning the three indirect pathways tested, the indirect effect through self-esteem (*X* → *M*_1_ → *Y*) was statistically significant [*β* = .011, 95% CI (0.000, 0.029)]; indicating that athletes with higher self-esteem exhibited reduced susceptibility effects on doping intention. Meanwhile, the indirect effect through self-efficacy (*X* → *M*_2_ → *Y*), although *X* did not significantly predict *M*_2_, was overall significant and negative [*β* = –.002, 95% CI (–0.011, –0.004)], suggesting that lower susceptibility was associated with lower doping intention via increases in self-efficacy. For the last indirect pathway, the sequential mediation (*X* → *M*_1_ → *M*_2_ → *Y*), the full mediation chain was significant [*β* = .001, 95% CI (0.000, 0.003)], these results indicate that susceptibility are negatively related to self-esteem, which subsequently are positive associated with self-efficacy, ultimately reducing the positive association between susceptibility to doping with doping intention.

Regarding the specific objectives of the study, five sequential mediation models were tested in which sociodemographic variables (gender, age, years of experience, level of practice, and academic level) were evaluated as predictors (*X*) of doping intention (*Y*), mediated by self-esteem (*M*1) and self-efficacy (*M*2). The results show that only age has a significant total effect (*B* = −0.221, *p* = 0.008) and a direct effect (*B* = −0.166, *p* = 0.047) on doping intention (Table 5). However, a partial mediation model through self-esteem was observed with a significant indirect effect (*B* = −0.047, 95% CI = −0.091, −0.016). Self-efficacy alone and the sequential model did not have a significant indirect effect, as the confidence interval crossed zero. In the other models, neither the total effect of the model nor the direct effect was significant on doping intention. Self-esteem acts as a significant indirect effect between this association for gender (*B* = 0.032, 95% CI = 0.003, 0.071) and years of experience (*B* = −0.031, 95% CI = −0.053, −0.013).

**Table 5:** Direct and indirect effects of sociodemographic characteristics on doping intention via self-esteem and self-efficacy.

Model	*C* (p)	*C*’ (p)	*M*1 [IC 95%]	*M*2 [IC 95%]	*M*1 → *M*2 [IC 95%]	*R*^2^ (*Y*)
Model 1	*B* = −0.046 (.507)	*B* = −0.072 (.292)	*B* = 0.032 [0.003, 0.071]	*B* = −0.007 [−0.022, 0.004]	*B* = 0.002 [−0.001, 0.005]	0.001
Model 2	*B* = −0.221 (.008)	*B* = −0.166 (.047)	*B* = −0.047 [−0.091, −0.016]	*B* = −0.006 [−0.022, 0.005]	*B* = −0.002 [−0.007, 0.001]	0.018
Model 3	*B* = 0.003 (.889)	*B* = 0.035 (.163)	*B* = −0.031 [−0.053, −0.013]	*B* = 0.001 [−0.004, 0.006]	*B* = −0.001 [−0.004, 0.001]	0.000
Model 4	*B* = −0.070 (.313)	*B* = −0.066 (.334)	*B* = −0.011 [−0.043, 0.014]	*B* = 0.008 [−0.003, 0.027]	*B* = −0.001 [−0.003, 0.001]	0.003
Model 5	−0.043 (.259)	−0.026 (.491)	−0.013 [−0.032, 0.000]	−0.003 [−0.013, 0.003]	−0.001 [−0.002, 0.001]	0.003

Model 1: Sex → Self-esteem → Self-efficacy → Doping intention; Model 2: Age → Self-esteem → Self-efficacy → Doping intention; Model 3: Experience → Self-esteem → Self-efficacy → Doping intention; Model 4: Level (amateur/exercisers) → Self-esteem → Self-efficacy → Doping intention; Model 5: Academic level → Self-esteem → Self-efficacy → Doping intention; *B* = unstandardized coefficients; CI = 95% bootstrap confidence interval based on 5,000 samples. C: Total effect; C’: Direct effect; *M*_1_: Indirect effect X → Self-esteem → Doping intention; *M*_2_: Indirect effect *X* → Self-efficacy → Doping intention; *M*1 → *M*2: Sequential indirect effect *X* → Self-esteem → Self-efficacy → Doping intention; *R*^2^: *R* square of the final model (*Y*).

## 4 Discussion

The present study aimed to analyze the sequential mediational effect of self-esteem and self-efficacy over the influence of doping susceptibility on doping intention in exercisers. Despite previous research examining the relationship or influence of self-esteem and self-efficacy on doping behaviors, this study is one of the few that proves the capability of both psychological dimensions against doping behaviors, specifically in non-high performance or competitive athletes.

Based on the above, the results of the study show that the model supports a partial mediation structure: susceptibility to doping is related to doping intention both directly and indirectly through self-esteem and self-efficacy (although to a small magnitude). Self-esteem demonstrated the strongest mediating role, while self-efficacy contributed additional but comparatively smaller indirect effects. So, our first hypothesis was confirmed “self-esteem and self-efficacy will jointly mediate the relationship between doping susceptibility and doping intention among competitive and recreational athletes”. According to the sequential mediation model employed, the results highlight that doping susceptibility has a positive relationship with doping intention but, self-esteem and self-efficacy indirectly help to reduce this relationship even when subjects are susceptible to dope. The main findings support empirical evidence with respect to the relevance of self-esteem and self-efficacy as protectors' psychological mechanisms against doping susceptibility and intention (24, 48, 63). These findings highlight the importance of psychological resources in attenuating the pathway from susceptibility to the intention to engage in doping behaviors.

As second hypothesis the study postulates that “self-esteem will mediate the relationship between doping susceptibility and doping intention”. Our results support the hypothesis, where self-esteem mediates negatively the relationship of susceptibility to dope with doping intention of the participants, representing a possible protector function at risk situations or behaviors related to doping, agreeing with results provided by previous research (36, 39). Due to the limited evidence on this topic in comparable samples, the present findings are supported by a study that evaluated coaches' perceptions over the relevance of self-esteem as barrier to prevent the risk to engagement on doping behaviors (63). Coaches reveal that athletes with stronger self-esteem are less susceptibility to engage in doping because they perceive themselves as sure of their capabilities. On the other hand, those who showed less self-esteem are more prone to surrender to external pressures, guiding their behaviors to take impulsive and moral disengagement decisions (64). Therefore, promoting healthier and stronger self-esteem will help exercisers to deal with external pressures such body stereotypes, comparison with others and social validation need, allowing them get satisfied with their own progress and well-being (34); this, in turn is related to the second psychological mechanism evaluated in the present study, self-efficacy.

The last hypothesis of the study “self-efficacy will mediate the relationship between doping susceptibility and doping intention” showed that this variable attenuates (lower than self-esteem) the intention to dope when the subject has more susceptibility to dope, coinciding with many studies which had previously proved the self-efficacy mediation effect on doping behaviors (24, 46, 48). As self-esteem, self-efficacy is conceived as an armor against risk behaviors for health and quality of life (41). Bandura (43) defines it as the capability to persist over the obstacles throughout a solid system of self-belief supporting the adaptative coping behaviors. Thus, self-efficacy inhibits the probability to engage in doping behaviors by the personal ability to self-control and resist against temptations to look for shortcuts in the pursuit of the goals (29, 32).

On the other hand, in athletes had been reported that lower self-efficacy is reinforced by cognitions such fear of failure (24) and defeats experiences, which can be triggers of pessimistic beliefs, driving them to fall into pro-doping behaviors easily (46). For this reason, self-efficacy is conceived as barrier against doping intention and use in athletes (48). In addition, who perceived themselves with higher mental toughness and confidence have less difficulty to restructure their beliefs when the context push them to dope and promotes moral disengagement normalizing these risk behavior (9).

Asking to the specific study aim, sociodemographic variables association with doping intention and the self-esteem and self-efficacy mediation quality were tested. The results indicate that age is significantly associated with doping intention, both directly and indirectly through self-esteem. Older participants showed higher levels of self-esteem and lower doping intention, while younger participants had lower levels of self-esteem and a greater willingness to dope. From an evolutionary development perspective, the findings are consistent with models describing a process of progressive consolidation of identity, self-regulation, and emotional stability throughout the life cycle. During young adulthood, individuals are often in stages of exploration, professional establishment, and consolidation of personal goals, which may involve greater sensitivity to external evaluation, social comparison, and pressure to achieve (65). These dynamics could explain the relatively lower levels of self-esteem observed in this group and their greater vulnerability to performance-oriented instrumental behaviors, such as doping (32). The fact that the relationship between age and doping intention is partially mediated by self-esteem suggests that the development of self-worth is a relevant explanatory mechanism for age differences (24, 47). In other words, it is not only chronological age that reduces doping intention, but also the psychological changes associated with development that influence self-perception.

On the other hand, the results show that there are no significant association of sex with doping intention, however, significant indirect effect was identified through self-esteem. Previous literature that has documented gender differences in self-esteem, particularly in sports contexts where performance standards, body image, and social comparison can differentially influence men and women. The lower self-esteem observed in women could be associated with sociocultural and contextual factors that affect the perception of self-worth in competitive environments (66). Although gender did not directly predict doping intention, it did so indirectly through self-esteem. Self-esteem showed a significant negative effect on doping intention, indicating that higher levels of self-esteem are associated with a lower willingness to take doping substances (39).

Similarly to sex, although the results show that years of practice do not have a direct effect on the intention to dope, they do have a significant indirect effect through self-esteem. Specifically, longer practice time is associated with higher levels of self-esteem, which in turn is related to a lower intention to dope. From the perspective of sports identity development, prolonged practice not only involves the accumulation of technical skills, but also the progressive construction of an identity linked to the role of athlete. Sports identity is consolidated through repeated experiences of competition, overcoming challenges, and social recognition, which contributes to a more stable and coherent self-perception (67). In this sense, literature suggests that athletes with more experience have developed a more integrated identity and a more solid self-assessment. This consolidation of identity could explain the significant increase in self-esteem observed as the years of practice increase. Conversely as a risk factor, those in the early stages (less than one or two years of practice) may experience greater instability in their athletic self-concept, which could increase their vulnerability to performance-oriented instrumental strategies, such as doping (48).

Following to explain the other results of the study, high self-reported levels of self-esteem and self-efficacy as well as lower levels of doping susceptibility and intention are related to the sample characteristics of people who practice exercise for health and leisure. When people are engaged to exercise and sport with regularity, they experiment the physical and mental benefits over their health and enhance their well-being perceptions (68), increasing their self-esteem and self-efficacy. While, in this kind of population, the external (social) and internal (personality) pressures are different than competitive athletes (69), who experiment high performance demands to gain results and social recognition, as well as faster recovery process and higher opportunity to obtain better economic rewards and sponsorship, increasing the circumstances related to higher susceptibility to engage in doping (5, 70).

Although the susceptibility and intention to dope are relatively low in the sample studied, this results are in line with other studies (24, 60) who evidenced percentages up to 30% considering from little to a lot of intention (in our sample, doping susceptibility and intention were from 4.8% to 21.4% and 10.2 to 34.7% respectively). According to the Social Science Research Package for Anti-Doping Organizations (55), it is important consider those athletes or people who have at least a little consideration to use prohibited substances for enhance their performance or image. Those subjects are propense to develop several clinical damages. In addition, in the specific case of our study, participants represent a particular sub-population where higher rates of doping intention or susceptibility as had been reported in previous studies (14, 71). Outside of federated sports, the use of performance and image enhancing drugs is recognized as an emerging phenomenon associated with performance goals and body image, with reports of anabolic-androgenic steroid use among gym users (72). Finally, it should be considered that, in active populations, the widespread use of supplements may increase the risk of exposure to prohibited substances (including inadvertent exposure), so monitoring “trends” and early signs (attitudes, susceptibility, and intention) is key as a public health and sports integrity strategy (73).

Finally, other sociocultural factors in the incidence of doping that were not considered in this study are recognized, for example, erroneous beliefs, a sports culture focused on results, lack of education about the risks of taking doping substances, and the importance of conscious and responsible supplementation (55). The aim is to consider addressing this in future lines of research.

### 4.1 Study limitations and practical implications

As any study, the present research results are not exempt from limitations**.** First, the nature of a cross-sectional design restricts the possibility to estimate causal relationships between variables throughout a period. Even sequential mediation analysis showed promising results, it can be interpreted with caution, because, although they were statistically significant, they were small in magnitude. In addition, the use of self-reported measures represents some risk error due to the influence of social desirability and/or the subjective interpretations of the self-perception of the participants, so, the psychological behavior observed must be carefully read (self-esteem, self-efficacy and doping susceptibility and intention). Nevertheless, the study employed the instrument recommended to evaluate the phenomenon of doping behaviors and psychological dimensions considering the anonymity of the participants and the use of hypothetical cases to attenuate social desirability and the risk to be marked or punished in case of being on favor or report to uses doping substances. Because of the diversity of the types of exercises practiced by the participants, as well as the sampling method used, the authors acknowledge that there is a bias in the selection of participants, with a possible overrepresentation in terms of educational level and connection with coaches from affiliated sports clubs, gyms or fitness centers. The authors also recognize that the wide age range of the sample is a limitation. However, this is justified given that literature establishes a certain risk of unintentional doping in recreational athletes, a population that has been little studied and requires greater attention. Due to this lack of data on exercising populations of different ages, the authors decided to represent the most likely ages based on this initial approach to the study. Consequently, it was not possible to perform an analysis by type of sport or age due to the heterogeneity of the sample and the disproportionate size of the subgroups. Given the above, it should be recognized that the results should not be generalized and that conclusions should be drawn with caution. Therefore, it is necessary to expand this research line, developing more complex studies, such as longitudinal follow-ups, to determine the mechanism by which self-esteem and self-efficacy influence doping behavior. Authors also recognize the limited scope of the psychological variables analyzed. The literature has reported that variables such as motivational climate and morality are relevant to understanding doping behavior, but the study was aimed at understanding the influence of intrapersonal variables like self-esteem and self-efficacy.

Regarding the pointed limitations, the results of the study are relevant providing evidence and visibility of a population with less attention but with high risk to get into risk behaviors for their health with the consumption of supplements and doping substances. Competitive or professional athletes usually have experts as support personnel to help and orient them to enhance their performance, but, amateur athletes and people who practice exercise are limited to access to the professional health support, nevertheless, the accessibility to buy and take all the options for enhancing performance or body composition put them into a high risk for their health. So, this study is helping to reduce this research gap.

Sports psychologists and health institutions should work on developing group psychoeducational workshops focused on programs to improve self-esteem, training in coping skills, and self-confidence to prevent the use of risky and/or illegal substances. In addition, training modules focused on coaches should be developed with the aim of fostering supportive motivational climates. According to a recent meta-analysis on the effectiveness of educational interventions to prevent doping in athletes (25), these types of programs should be structured in short periods (4–6 weeks), with the active participation of athletes and the integration of cognitive and affective domains.

## 5 Conclusions

At individual level, self-esteem seems to be a stronger mediator of the doping susceptibility effect over the intention to dope. Together, the sequential influence of self-esteem and self-efficacy over doping susceptibility and intention relationship reflects a more powerful mediational process. In addition, although the risk level refers to the doping susceptibility and intention in the subjects of the study, it requires special attention at prevention level in similar context which evidence is limited. In general, it can be concluded that the lower levels of pro-doping behaviors are related to the high levels of self-esteem and self-efficacy, being mechanisms of protection against doping susceptibility and intention reinforcing the exercisers health. The results of the present study remark the need for work on educational interventions for doping prevention throughout the empowerment of psychological dimensions such self-esteem and self-efficacy. Following anti-doping agency standards, training is required for personnel involved in managing athletes: psychologists, coaches, nutritionists, therapists, and doctors. The aim is to promote doping prevention, identifying the values of clean sport, ethics in sport, and awareness of health risks in order to develop critical thinking, thereby strengthening psychological skills and implementing workshops designed for psychoeducation in different sports and educational settings according to sport level (recreational, amateur, elite).

## Data Availability

The raw data supporting the conclusions of this article will be made available by the authors, without undue reservation.

## References

[1] Gómez Píriz PT. El Entrenamiento Deportivo en el Siglo XXI [Sports Training in the 21st Century]. Alcalá la Real, Jaén: Formación Alcalá (2011). p. 218.

[2] Pennington CG. Moral development and sportsmanship in physical education and sport. J Phys Educ Recreat Dance. (2017) 88(9):36–42. 10.1080/07303084.2017.1367745

[3] Engelberg T Moston S Skinner J. The final frontier of anti-doping: a study of athletes who have committed doping violations. Sport Manag Rev. (2015) 18(2):268–79. 10.1016/j.smr.2014.06.005

[4] Filleul V d’Arripe-Longueville F Pavot D Bimes H Maillot J Meinadier E Doping in elite cycling: a qualitative study of the underlying situations of vulnerability. Front Sports Act Living. (2024) 6:1482103. 10.3389/fspor.2024.1482103 39650252 PMC11620872

[5] Morente-Sánchez J Zabala M. Doping in sport: a review of elite Athletes’ attitudes, beliefs, and knowledge. Sports Med. (2013) 43(6):395–411. 10.1007/s40279-013-0037-x 23532595

[6] Backhouse SH Whitaker L Petróczi A. Gateway to doping? Supplement use in the context of preferred competitive situations, doping attitude, beliefs, and norms. Scand J Med Sci Sports. (2013) 23(2):244–52. 10.1111/j.1600-0838.2011.01374.x 22092778

[7] Soberanes KM Frías EC Balandrán LR Fermán MER Méndez NM Velasco-Bejarano B. Sustancias dopantes y su incidencia: una visión retrospectiva del laboratorio nacional de prevención y control del dopaje de méxico [doping substances and their incidence: a retrospective view from Mexico’s national laboratory for doping prevention and control]. Adicciones. (2019) 31(3):201–11. 10.20882/adicciones.1012 30059594

[8] Gleaves J Petróczi A Folkerts D De Hon O Macedo E Saugy M Doping prevalence in competitive sport: evidence synthesis with “best practice” recommendations and reporting guidelines from the WADA working group on doping prevalence. Sports Med. (2021) 51(9):1909–34. 10.1007/s40279-021-01477-y 33900578

[9] Boardley ID Smith AL Mills JP Grix J Wynne C. Empathic and self-regulatory processes governing doping behavior. Front Psychol. (2017) 8:1495. 10.3389/fpsyg.2017.01495 29018370 PMC5614971

[10] European Commission. Study on Doping Prevention. A map of Legal, Regulatory and Prevention Practice Provisions in EU 28. Luxembourg: Publications Office of the European Union (2014). p. 154. Available online at: https://data.europa.eu/doi/10.2766/86776

[11] García-Arnés JA García-Casares N. Doping and sports endocrinology: anabolic-androgenic steroids. Rev Clínica Esp Engl Ed. (2022) 222(10):612–20. 10.1016/j.rceng.2022.09.003 36400345

[12] Henning A. Challenges to promoting health for amateur athletes through anti-doping policy. Drugs Educ Prev Policy. (2017) 24(3):306–13. 10.1080/09687637.2016.1208732 PMC5515538 28736489

[13] Lazuras L Barkoukis V Loukovitis A Brand R Hudson A Mallia L Corrigendum: “I want it all, and I want it now”: lifetime prevalence and reasons for using and abstaining from controlled performance and appearance enhancing substances (PAES) among young exercisers and amateur athletes in five European countries. Front Psychol. (2018) 9:1162. 10.3389/fpsyg.2018.01162 30083116 PMC6069581

[14] Christiansen AV Frenger M Chirico A Pitsch W. Recreational Athletes’ use of performance-enhancing substances: results from the first European randomized response technique survey. Sports Med Open. (2023) 9(1):1. 10.1186/s40798-022-00548-2 36617340 PMC9825800

[15] Amaral JMX Kimergård A Deluca P. Prevalence of anabolic steroid users seeking support from physicians: a systematic review and meta-analysis. BMJ Open. (2022) 12(7):e056445. 10.1136/bmjopen-2021-056445 35788077 PMC9255415

[16] Hoseini R Hoseini Z. Exploring the prevalence of anabolic steroid use among men and women resistance training practitioners after the COVID-19 pandemic. BMC Public Health. (2024) 24(1):798. 10.1186/s12889-024-18292-5 38481173 PMC10938795

[17] Pereira E Moyses SJ Ignácio SA Mendes DK Silva DSDA Carneiro E Prevalence and profile of users and non-users of anabolic steroids among resistance training practitioners. BMC Public Health. (2019) 19(1):1650. 10.1186/s12889-019-8004-6 31818274 PMC6902556

[18] Ruwuya J Juma BO Woolf J. Challenges associated with implementing anti-doping policy and programs in Africa. Front Sports Act Living. (2022) 4:966559. 10.3389/fspor.2022.966559 36570497 PMC9772459

[19] Aguilar M Muñoz-Guerra J Plata MDM Del Coso J. Thirteen years of the fight against doping in figures. Drug Test Anal. (2017) 9(6):866–9. 10.1002/dta.2168 28105782

[20] Garthe I Maughan RJ. Athletes and supplements: prevalence and perspectives. Int J Sport Nutr Exerc Metab. (2018) 28(2):126–38. 10.1123/ijsnem.2017-0429 29580114

[21] Hurst P. Are dietary supplements a gateway to doping? A retrospective survey of Athletes’ substance use. Subst Use Misuse. (2023) 58(3):365–70. 10.1080/10826084.2022.2161320 36645808

[22] Piacentino D Kotzalidis GD Del Casale A Aromatario MR Pomara C Girardi P Anabolic-androgenic steroid use and psychopathology in athletes. A systematic review. Curr Neuropharmacol. (2015) 13(1):101–21. 10.2174/1570159X13666141210222725 26074746 PMC4462035

[23] Nelson BS Hildebrandt T Wallisch P. Anabolic–androgenic steroid use is associated with psychopathy, risk-taking, anger, and physical problems. Sci Rep. (2022) 12(1):9133. 10.1038/s41598-022-13048-w 35650220 PMC9160254

[24] Blank C Schobersberger W Leichtfried V Duschek S. Health psychological constructs as predictors of doping susceptibility in adolescent athletes. Asian J Sports Med. (2016) 7(4):e35024. 10.5812/asjsm.35024 28144408 PMC5256272

[25] Reynoso-Sánchez LF Molgado-Sifuentes A Muñoz-Helú H López-Walle JM Soto-García D. Effective intervention features of a doping prevention program for athletes: a systematic review with meta-analysis. Sports. (2025) 13(4):108. 10.3390/sports13040108 40278734 PMC12031626

[26] Mudrak J Slepicka P Slepickova I. Sport motivation and doping in adolescent athletes. PLoS One. (2018) 13(10):e0205222. 10.1371/journal.pone.0205222 30286200 PMC6171920

[27] Mooney R Simonato P Ruparelia R Roman-Urrestarazu A Martinotti G Corazza O. The use of supplements and performance and image enhancing drugs in fitness settings: a exploratory cross-sectional investigation in the United Kingdom. Hum Psychopharmacol. (2017) 32(3):e2619. 10.1002/hup.2619 28657184

[28] Boardley ID Grix J Harkin J. Doping in team and individual sports: a qualitative investigation of moral disengagement and associated processes. Qual Res Sport Exerc Health. (2015) 7(5):698–717. 10.1080/2159676X.2014.992039

[29] Ring C Kavussanu M. The role of self-regulatory efficacy, moral disengagement and guilt on doping likelihood: a social cognitive theory perspective. J Sports Sci. (2018) 36(5):578–84. 10.1080/02640414.2017.1324206 28481691

[30] Attlee A Haider A Hassan A Alzamil N Hashim M Obaid RS. Dietary supplement intake and associated factors among gym users in a university community. J Diet Suppl. (2018) 15(1):88–97. 10.1080/19390211.2017.1326430 28557663

[31] Muñoz Maldonado GE Gómez Renaud VM Garza Ocañas L Badillo Castañeda CT. Suplementos deportivos: ¿riesgo a la salud? [sports supplements: a health risk? Biotecnia. (2022) 24(1):122–32. 10.18633/biotecnia.v24i1.1557

[32] Ntoumanis N Ng JYY Barkoukis V Backhouse S. Personal and psychosocial predictors of doping use in physical activity settings: a meta-analysis. Sports Med. (2014) 44(11):1603–24. 10.1007/s40279-014-0240-4 25138312

[33] Wilkins L Dunn A Zoob Carter BN Boardley ID. Exploring the relationship between mindset and psychological factors linked to doping. Perform Enhanc Health. (2022) 10(4):100238. 10.1016/j.peh.2022.100238

[34] Branden N. The Six Pillars of Self-Esteem. New York: Bantam (1995). p. 368.

[35] Babiss LA Gangwisch JE. Sports participation as a protective factor against depression and suicidal ideation in adolescents as mediated by self-esteem and social support. J Dev Behav Pediatr. (2009) 30(5):376–84. 10.1097/DBP.0b013e3181b33659 19692930

[36] Martínez-Casanova E Molero-Jurado MDM Pérez-Fuentes MDC. Self-Esteem and risk behaviours in adolescents: a systematic review. Behav Sci. (2024) 14(6):432. 10.3390/bs14060432 38920764 PMC11201250

[37] González-Hernández J Barrera-Vázquez D Gómez-López M. Self -Confidence in young athletes: a protective factor against perfectionism and anxiety in competitive grassroots sport. Percept Mot Skills. (2024) 131(6):2324–45. 10.1177/00315125241290563 39379131

[38] Simkin H Pérez-Marín M. Personalidad y autoestima: un análisis sobre el importante papel de sus relaciones [Personality and self-esteem: an analysis of the important role of your relationships]. Ter Psicológica. (2018) 36(1):19–25. 10.4067/s0718-48082017000300015

[39] Fuentes MC Garcia OF Garcia F. Protective and risk factors for adolescent substance use in Spain: self-esteem and other indicators of personal well-being and ill-being. Sustainability. (2020) 12(15):5962. 10.3390/su12155962

[40] Amr RA Al-Smadi AM Deiranieh RA Amr RA Mayyas AH Akasheh RT. Understanding the association of self-efficacy, mood, and demographics with physical activity in Syrian and Iraqi refugees: a cross-sectional study in Jordan. Sci World J. (2023) 2023:8876254. 10.1155/2023/8876254 PMC10522438 37766862

[41] Medrano-Ureña MDR Ortega-Ruiz R Benítez-Sillero JD. Physical fitness, exercise self-efficacy, and quality of life in adulthood: a systematic review. Int J Environ Res Public Health. (2020) 17(17):6343. 10.3390/ijerph17176343 32878182 PMC7504332

[42] Young MD Plotnikoff RC Collins CE Callister R Morgan PJ. Social cognitive theory and physical activity: a systematic review and meta-analysis. Obes Rev. (2014) 15(12):983–95. 10.1111/obr.12225 25428600

[43] Bandura A. Self-Efficacy: The Exercise of Control. New York: WH Freeman (1997). p. 604.

[44] Herzog-Krzywoszanska R Potoczny W Krzywoszanski LO. 4.5-10 Self-control and self-efficacy positively influence physical activity levels. Eur J Public Health. (2023) 33(Suppl 1):ckad133.218. 10.1093/eurpub/ckad133.218

[45] Ortiz-Romero M Garrido Guzmán ME Castañeda Vázquez C. Autoeficacia y resiliencia: diferencias entre deportistas practicantes de fitness/culturismo y no deportistas [Self-efficacy and resilience: differences between fitness/bodybuilding athletes and non-athletes]. Retos. (2021) 44:232–41. 10.47197/retos.v44i0.88937

[46] Barkoukis V Lazuras L Tsorbatzoudis H Rodafinos A. Motivational and social cognitive predictors of doping intentions in elite sports: an integrated approach. Scand J Med Sci Sports. (2013) 23(5):e330–40. 10.1111/sms.12068 23574429

[47] Lucidi F Zelli A Mallia L Grano C Russo PM Violani C. The social-cognitive mechanisms regulating adolescents’ use of doping substances. J Sports Sci. (2008) 26(5):447–56. 10.1080/02640410701579370 18274942

[48] Ntoumanis N Dølven S Barkoukis V Boardley ID Hvidemose JS Juhl CB Psychosocial predictors of doping intentions and use in sport and exercise: a systematic review and meta-analysis. Br J Sports Med. (2024) 58(19):1145–56. 10.1136/bjsports-2023-107910 38925889 PMC11503166

[49] Focaroli V Chiaro M Battaglia MV Guidetti L Velardi A. Educational intervention on awareness of health-damaging behaviors in educators. Sports. (2024) 12(12):348. 10.3390/sports12120348 39728888 PMC11679347

[50] Galli F Chirico A Codella R Zandonai T Deplano V Maria AD “I am on top!”: an interactive intervention program to promote self-regulation processes in the prevention of the use of doping in sports high schools. Eur J Investig Health Psychol Educ. (2023) 13(11):2630–41. 10.3390/ejihpe13110183 37998073 PMC10670151

[51] Hayes AF Rockwood NJ. Regression-based statistical mediation and moderation analysis in clinical research: observations, recommendations, and implementation. Behav Res Ther. (2017) 98:39–57. 10.1016/j.brat.2016.11.001 27865431

[52] Meltzoff J Cooper H. Critical Thinking About Research: Psychology and Related Fields. 2nd ed. Washington: American Psychological Association (2018). p. 541.

[53] Xi W Hinton A Lu B Krotki K Keller-Hamilton B Ferketich A Analysis of combined probability and nonprobability samples: a simulation evaluation and application to a teen smoking behavior survey. Commun Stat Simul Comput. (2024) 53(7):3285–301. 10.1080/03610918.2022.2102181 39184174 PMC11343077

[54] Tavares ASR Rosado AF Marôco J Calmeiro L Serpa S. Determinants of the intention to use performance-enhancing substances among Portuguese gym users. Front Psychol. (2020) 10:2881. 10.3389/fpsyg.2019.02881 32010010 PMC6971192

[55] Donovan R Jalleh G Gucciardi D. Research Package for Anti-Doping Organizations. Montreal: World Anti-Doping Agency (2015). p. 135.

[56] Secretaría de Gobernación. Reglamento de la ley General de Salud en Materia de Investigación Para la Salud [regulations of the General Health law on Health Research]. Ciudad de México: Diario Oficial de la Federación (2014). p. 31. Available online at: https://dof.gob.mx/index.php?year=2014&month=12&day=30

[57] World Medical Association. World medical association declaration of Helsinki: ethical principles for medical research involving human subjects. JAMA. (2025) 333(1):71–4. 10.1001/jama.2024.21972 39425955

[58] Guelmami N Ben Ezzeddine L Hatem G Trabelsi O Ben Saad H Glenn JM The ethical compass: establishing ethical guidelines for research practices in sports medicine and exercise science. Int J Sport Stud Health. (2024) 7(2):31–46. 10.61838/kman.intjssh.7.2.4

[59] Gucciardi DF Jalleh G Donovan RJ. Does social desirability influence the relationship between doping attitudes and doping susceptibility in athletes? Psychol Sport Exerc. (2010) 11(6):479–86. 10.1016/j.psychsport.2010.06.002

[60] Guo L Liang W Baker JS Mao ZX. Perceived motivational climates and doping intention in adolescent athletes: the mediating role of moral disengagement and sportspersonship. Front Psychol. (2021) 12:611636. 10.3389/fpsyg.2021.611636 33841245 PMC8024559

[61] Rosenberg M. Society and the Adolescent Self-Image. Princenton (NJ): Princenton University Press (1965). p. 338.

[62] Reynoso-González OU Valdés-García KP Santana-Campas MA De Luna-Velasco LE Hermosillo-De La Torre AE. Psychometric properties of the Rosenberg self-esteem scale in Mexican university students. Acta Univ. (2022) 32:1–12. 10.15174/au.2022.3441

[63] Nicholls AR Perry JL Levy AR Meir R Jones L Baghurst T Coach perceptions of performance enhancement in adolescence: the sport drug control model for adolescent athletes. Perform Enhanc Health. (2014) 3(2):93–101. 10.1016/j.peh.2015.07.001

[64] Kang S Kim I Lee K. Predicting deviant behaviors in sports using the extended theory of planned behavior. Front Psychol. (2021) 12:678948. 10.3389/fpsyg.2021.678948 34566759 PMC8459903

[65] Crocetti E Albarello F Meeus W Rubini M. Identities: a developmental social-psychological perspective. Eur Rev Soc Psychol. (2022) 34(1):161–201. 10.1080/10463283.2022.2104987 38504829 PMC10950040

[66] Morano M Robazza C Ruiz MC Cataldi S Fischetti F Bortoli L. Gender-Typed sport practice, physical self-perceptions, and performance-related emotions in adolescent girls. Sustainability. (2020) 12(20):8518. 10.3390/su12208518

[67] Vera Ortega DE Mayorga Capa DI Iñaguazo Jordan SV Atiencia Armijos PA. Relación entre la práctica deportiva y la autoestima en niños, niñas y adolescentes: una revisión sistemática. SAGA Rev Científica Multidiscip. (2025) 2(1):88–102. 10.63415/saga.v2i1.34

[68] Sarr DC. Self-Esteem, psychological factors and sports participation: a comparative study among basketball players and non-active individuals. Int J Multidiscip Res. (2025) 7(2):1–6. 10.36948/ijfmr.2025.v07i02.38746

[69] Kuettel A Larsen CH. Risk and protective factors for mental health in elite athletes: a scoping review. Int Rev Sport Exerc Psychol. (2020) 13(1):231–65. 10.1080/1750984X.2019.1689574

[70] Terreros JL Manonelles P López-Plaza D. Relationship between doping prevalence and socioeconomic parameters: an analysis by sport categories and world areas. Int J Environ Res Public Health. (2022) 19(15):9329. 10.3390/ijerph19159329 35954686 PMC9367925

[71] Hamid AA Alomani L Aljuresan A Alahmad W Alluwaim Z. Steroid and illicit drug abuse in the health and fitness community: a systematic review of evidence. Emerg Trends Drugs Addict Health. (2025) 5:100172. 10.1016/j.etdah.2025.100172

[72] Arsovski D Amedovski H. Use of legal and illegal performance enhancing drugs and supplementsin gym users: prevalence, risks, and satisfaction. Hrvat Šport Vjesn. (2025) 40(1):50–8. 10.69589/hsv.40.1.5

[73] World Anti-Doping Agency. A Systemic Risk Assessment of Inadvertent Doping Through Supplement use. Montreal: World Anti-Doping Agency (2025). Available online at: https://www.wada-ama.org/en/resources/social-science-research/systemic-risk-assessment-inadvertent-doping-through-supplement

